# Mutation of *FLNA* attenuating the migration of abdominal muscles contributed to Melnick–Needles syndrome (MNS) in a family with recurrent miscarriage

**DOI:** 10.1002/mgg3.2145

**Published:** 2023-02-03

**Authors:** Xin Luo, Zailin Yang, Jing Zeng, Jing Chen, Ningxuan Chen, Xiaoyan Jiang, Qinlv Wei, Ping Yi, Jing Xu

**Affiliations:** ^1^ Department of Obstetrics and Gynecology The Third Affiliated Hospital of Chongqing Medical University Chongqing China; ^2^ Chongqing University Cancer Hospital Chongqing China; ^3^ Department of Obstetrics and Gynecology Yubei District Chinese Medicine Hospital Chongqing China

**Keywords:** *FLNA*, Melnick–Needles syndrome, midline structure dysplasia, recurrent miscarriage

## Abstract

**Background:**

Filamin A, encoded by the X‐linked gene *FLNA*, links the cell membrane with the cytoskeleton and acts as a regulator of the actin cytoskeleton. Mutations in *FLNA* cause a large spectrum of congenital malformations during embryonic development, including Melnick–Needles syndrome (MNS). However, reports of MNS, especially in males, are rare, and the pathogenesis molecular mechanisms are not well understood.

**Methods:**

We found a family with two consecutive miscarriages of similar fetuses with multiple malformations. DNA was extracted from peripheral blood and tissues, and whole exome sequencing was performed for genetic analysis. Then, we created a C57BL/6 mouse with a point mutation by CRISPR/Cas‐mediated genome engineering. The migration of primary abdominal muscle cell was detected by wound healing assay.

**Results:**

The first fetus showed congenital hygroma colli and omphalocele identified by ultrasound at 12 wks; the second fetus showed hygroma colli and thoraco abdominoschisis at 12 wks, with a new hemizygous mutation c.4420G>A in exon 26 of the *FLNA* gene, which is predicted to cause an amino acid substitution (p.Asp1474Asn). The mother and grandmother were both present in the c.4420G>A heterozygous state, and the mother's healthy brother had wild‐type *FLNA*. These *FLNA*‐mutated mice exhibited a broader central gap between the rectus abdominis than the wild type (WT), similar to the midline structure dysplasia of the abdominal wall in the two fetuses. Wound healing assays showed the attenuated migration capacity of abdominal muscle cells in mice with mutated *FLNA*. Finally, we summarized the cases of MNS with *FLNA* mutation from the accessible published literature thus far.

**Conclusion:**

Our research revealed a mutation site of the *FLNA* for MNS and explored the mechanism of midline structure dysplasia in the abdominal wall of male patients, which could provide more evidence for the clinical diagnosis and genetic counseling of families with these disorders.

## Graphical abstract

Our research revealed a novel mutation site of the FLNA for MNS and explored the mechanism of midline structure dysplasia in the abdominal wall of males patients.

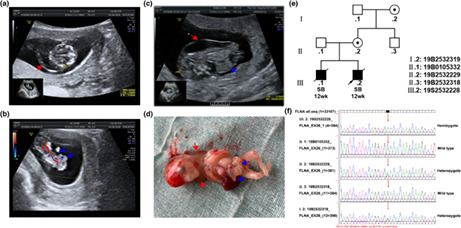

## 1 INTRODUCTION

The *FLNA* (OMIM: 300017) gene located on Xq28 encodes the filamin A protein by 48 exons (Clapham et al., 2012). Filamin A is a 280‐kD actin‐binding protein that possesses an N‐terminal actin‐binding domain (ABD) and an extended region comprised of 24 repeated immunoglobulin‐like (Ig‐like) domains, which regulate the actin cytoskeleton by interacting with transmembrane receptor‐binding regions (Kanaji et al., 2012). Mutations and dysfunction in the *FLNA* gene lead to a large spectrum of rare developmental disorders, generically including brain, bone, muscle, cardiovascular, and other malformations (Donada et al., 2019). Loss‐of‐function mutations in *FLNA* result in prenatal lethality in males and X‐linked periventricular nodular heterotopia (PNH) in females and are related to cardiovascular abnormalities (Clapham et al., 2012). Reduced *FLNA* levels could also cause a low platelet count by affecting proplatelet formation and RhoA activation (Donada et al., 2019). In contrast, gain‐of‐function variants could cause a series of skeletal dysplasias that are characterized by cleft palate, hyperostosis of the skull, anomalies of the tubular bones, which are mainly classified into otopalatodigital syndrome type I (OPD1, OMIM# 311300), otopalatodigital syndrome type II (OPD2, OMIM# 304120), fronto‐metaphyseal dysplasia 1 (FMD1, OMIM# 305620), Melnick‐Needles syndrome (MNS, OMIM# 309350), and terminal osseous dysplasia (TOD, OMIM# 300244) (Hehr et al., 2006).

MNS, an extremely rare skeletal anomaly belonging to one of the otopalatodigital spectrum disorders (OPDSD), is the most severe disease that causes a lethal phenotype in hemizygous males and skeletal dysplasia in heterozygous survivable females since gain‐of‐function mutations in *FLNA* (Naudion et al., 2016). Almost all patients are females, as males with MNS have skeletal anomalies as well as variable malformations in the brain, heart, intestines, and kidneys that usually lead to prenatal lethality (Santos et al., 2010). The general symptoms of MNS include craniofacial disproportion, prominent supraorbital ridge, exorbitism, hypodontia, micrognathia, scoliosis, short ribbon‐like ribs, bowing of the long bones and sclerosis of the base of the skull (O'Connell et al., 2018). Partially serious MNS patients present with hydronephrosis, mitral and tricuspid valve prolapse, hypoplastic thorax and omphalocele (Lykissas et al., 2013). Studies have revealed that MNS is an X‐linked dominant inheritance that is mostly caused by a missense mutation in exon 22 of the *FLNA* gene (Foley et al., 2010). However, the functions and mechanisms of mutation in *FLNA* remain elusive in the development of MNS.

In our research, we showed the mutation of *FLNA* for MNS spectrum disorder in a family in which two male fetuses acted as the probands and the mother and grandmother were germline carriers. The mutation c.4420G>A in exon 26 of the *FLNA* in this family has been previously documented, which could result in an amino acid Asp exchange to Asn. Moreover, we observed a wider central gap between the rectus abdominis in the homozygous and heterozygous groups than in the WT group, correlating with the decreased migration in abdominal muscle cells with mutation of *FLNA* in the mouse model and culturing primary cells. To this end, we collected previous studies and summarized the information of MNS cases carrying different *FLNA* mutation sites. Our observation indicates that mutation of *FLNA* might cause midline structure dysplasia in the abdominal wall in MNS male patients by reducing cell migration ability.

## 2 MATERIALS AND METHODS

### 2.1 Patient ascertainment

The family was ascertained through the clinical genetics service at the gynecology and obstetrics of the Third Affiliated Hospital of Chongqing Medical University. Informed consent was obtained from participants or their legal guardians before participation in this research. Blood samples of the family members and abnormal embryonic tissue from the proband were collected, and clinical photographs and fetal ultrasound imaging were obtained. This research obtained the ethical approval of the Ethics Committee of the Third Affiliated Hospital of Chongqing Medical University.

### 2.2 Animal experiments

Point mutations of *FLNA* (Homo sapiens: NM_001456.3; Mus musculus: NM_010227.3) mice were generated on the C57BL/6 background by Cyagen Biosciences Inc. using the CRISPR/Cas9 system. The gRNA to mouse *FLNA* gene, donor oligo containing corresponding D1474N (GAC to AAC) mutation sites and Cas9 mRNA were coinjected into fertilized mouse eggs to generate targeted knock‐in offspring. F2 founder animals were identified by PCR followed by sequence analysis and were bred to heterozygote mice to test germline transmission and F3 animal generation. Animal experiments were performed following guidelines approved by the Animal Ethics Committee of Chongqing Medical University. All mice were bred and maintained in the IVC room at the Laboratory Animals Center of Chongqing Medical University. The experiments were repeated three times with three mice per group.

### 2.3 Gene sequencing and analysis

The blood and tissue samples were sent to Beijing Genomics Institute (BGI, Shenzen, China), and whole exome sequencing (WES) was performed and analyzed at BGI.

### 2.4 Detection of the central gap between rectus abdominis

C57BL/6 female mice aged 16 weeks were killed by cervical dislocation and fixed on the dissecting table in the supine position. The skin of the abdomen was then separated with fine scissors to expose the abdominal muscles and the central gap between the rectus abdominis. The central gap was estimated with two methods: the first was the absolute distance, reflected by the direct measurement by digital caliper; the second was the relative distance, reflected by the ratio of the gap distance between rectus abdominis to the distance between epigastric arteries on both sides.

### 2.5 Primary abdominal muscle cell culture

Briefly, 8‐week‐old female mice were sacrificed by cervical dislocation and soaked for 5 minutes with 75% ethanol. Abdominal muscle was dissected and washed twice with sterile phosphate‐buffered saline (PBS). Then, tissues were sheared into small pieces and treated with 0.25% trypsin–EDTA (Solarbio, China) and collagenase type I (Solarbio, China) to digest into tissue debris or cell clumps. After a timely incubation, they were suspended in fresh DMEM (Gibco, USA) supplemented with 10% FBS (BI) and 1% penicillin/streptomycin (Solarbio, China). The cells were centrifuged and resuspended in complete medium and finally filtered through a 40‐mesh sterile nylon sieve. Primary cells were cultured at 37°C in 5% CO_2_ with humidity.

### 2.6 Cell migration assay

We utilized a wound healing assay to measure cell migration. Primary cells cultured between 10 and 15 days were seeded in 6‐well plates and incubated to almost full confluency. A wound was created using a 10 μL pipette tip in each well, washed twice with PBS and replaced with fresh medium. Images were obtained immediately under a microscope (Nikon, Japan) after scratching and were cultured in serum‐free medium for 24 h and photographed again for 48 h. The experiment was repeated independently three times. We measured the initial gap width (0 h as 100%) and the residual gap width at 24 and 48 h and evaluated the cell migration ability of the different groups.

### 2.7 Statistics

Statistical analyses and graphical presentation were performed with GraphPad Prism software 6.0. The results are presented as the mean ± SD and evaluated using an unpaired Student's t‐test (*p*‐value < 0.05 was considered to be significant).

## 3 RESULTS

### 3.1 Patient cohort in the family with recurrent miscarriage

**FIGURE 1:**
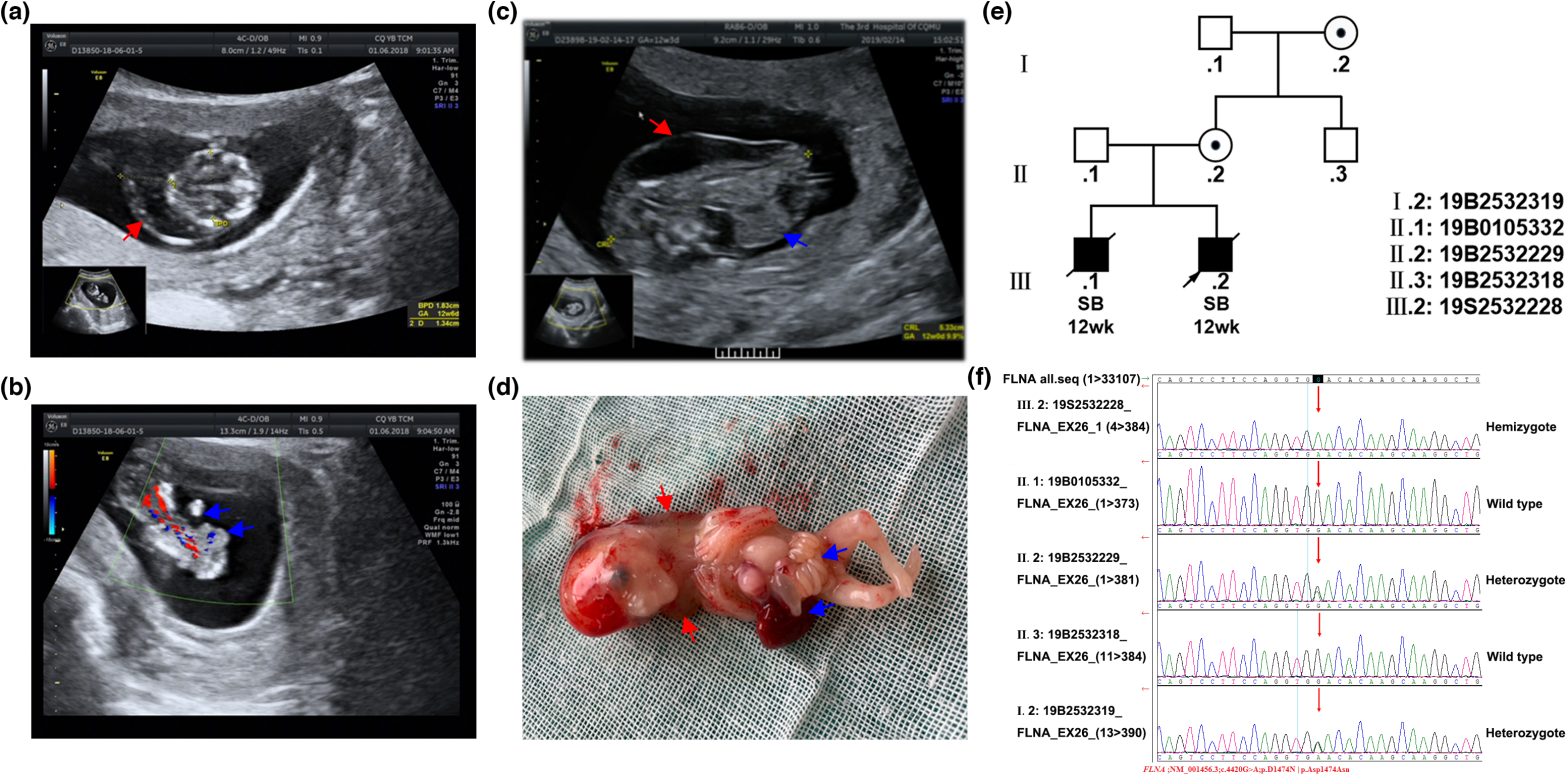
Ultrasonic examination and phenotype of the proband and his family genetic characteristics. (a, b) Ultrasonic examination of the first fetus (III.1) demonstrating hygroma colli (red arrow), omphalocele and unclear lower limbs (blue arrow). (c, d) Ultrasonic examination and phenotype of the second fetus (III.2) demonstrating hygroma colli (red arrow), thoracoceloschisis with visceral valgus (blue arrow) and abnormal lower limbs. (e) The genetic spectrum for this family with *FLNA* mutation; SB means stillbirth. (f) The mutational locus of *FLNA* (c.4420G>A) in this family by WES.

The family with a healthy nonconsanguineous couple came to our obstetrics and gynecology genetic consultation clinic. The mother (II.2) was 26 years old at the 12th week of pregnancy, denied any drug or alcohol use, and showed no obvious appearance deformities except micrognathia and exorbitism. Eight months ago, her first pregnancy ended in miscarriage at 12 weeks because the fetus (III.1) had severe malformations identified by ultrasound, including a hygroma colli, omphalocele and unclear lower limbs (Figure 1a,b). Copy number variation (CNV) detection was conducted after induction. The result showed that the patient was male, and no pathogenicity CNV could be found. Then, no further genetic examination was carried out. Unfortunately, when we performed an ultrasound examination for her second fetus, the fetus (III.2, 12 weeks) showed similar multiple congenital malformations, including a hygroma colli, thoracoceloschisis with visceral valgus and abnormal lower limbs (Figure 1c). The mother required miscarriage with medicine, and the lethiferous fetus was discharged through the vagina. There was a slightly translucent body with thoraco abdominoschisis covered by internal organs containing part of the umbilical cord, heart, lung, liver and intestine. He had hygroma colli on the back, his facial features looked like protuberant eyes, broad nasal bridge, micrognathia, full anterior fontanelle and the formed fingers are normal on each limb, the lower extremities were dysplasia and flexion deformities (Figure 1d).

### 3.2 Identification of mutations in the family

We detected CNV in the second fetus (III.2) and found that the sex was also male and without pathogenic CNV. We obtained consent for detailed clinical genetic surveys in this family and collected the tissue of the proband (III.2) and blood samples of his parents (II.1/II.2) to conduct WES. WES identified a hemizygous mutation on exon 26 of *FLNA* (c.4420G>A) in the proband and a heterozygous mutation of *FLNA* in the mother (II.2). The mutation was predicted to cause an amino acid substitution (p.Asp1474Asn). The father was identified as a wild type. Furthermore, *FLNA* mutation verification of the lineal relatives, including the uncle (II.3) and grandmother (I.2), was performed. The results showed that the grandmother (also with micrognathia and exorbitism) was a heterozygous carrier with *FLNA* mutation in the same locus and that the uncle was wild type (Figure 1f). According to the above findings, we presume III.1 as a hemizygote of *FLNA* mutation and draw the genetic map for this family (Figure 1e). In addition, it is worth mentioning that other heterozygous mutations with the proband have been identified, including *C5orf42* (NM_023073.3; c.7978C>T; p.Arg2660*), *TTC21B* (NM_024753.4; c.2251C>G; p.Gln751Glu), which could strengthen the contribution to this disease.

According to the joint consensus recommendation of the American College of Medical Genetics and Genomics and the Association for Molecular Pathology about the standards and guidelines for the interpretation of sequence variants, the mutation of *FLNA* (c.4420G>A) was considered the “uncertain significance”(Richards et al., 2015). However, using the 1000 Genomes Project as a reference, the frequency of mutation of this locus in the population is extremely low (<0.01) and demonstrates high evolutionary conservation (Best et al., 2021). For analysis of the p. Asp1474Asn, we used Polyphen software to predict the possible impact of an amino acid substitution on the structure and function of human proteins, and the results showed a harmful effect. Furthermore, the diseased phenotype of the proband male fetuses is highly related to the MNS, and the diseased phenotype cosegregated with the mutation in this family, manifested by lethal hemizygous males with severe malformations, surviving wild‐type males and heterozygous females with characteristic faces. The contribution of other mutations of *FLNA* exon 26 to MNS has been reported (Adzhubei et al., 2010; Robertson, 1993). Therefore, we speculated that the mutation of *FLNA* with our research is the main cause of the recurrent malformations in the family.

### 3.3 The corresponding FLNA mutation induced the malignant phenotype in mice

**FIGURE 2:**
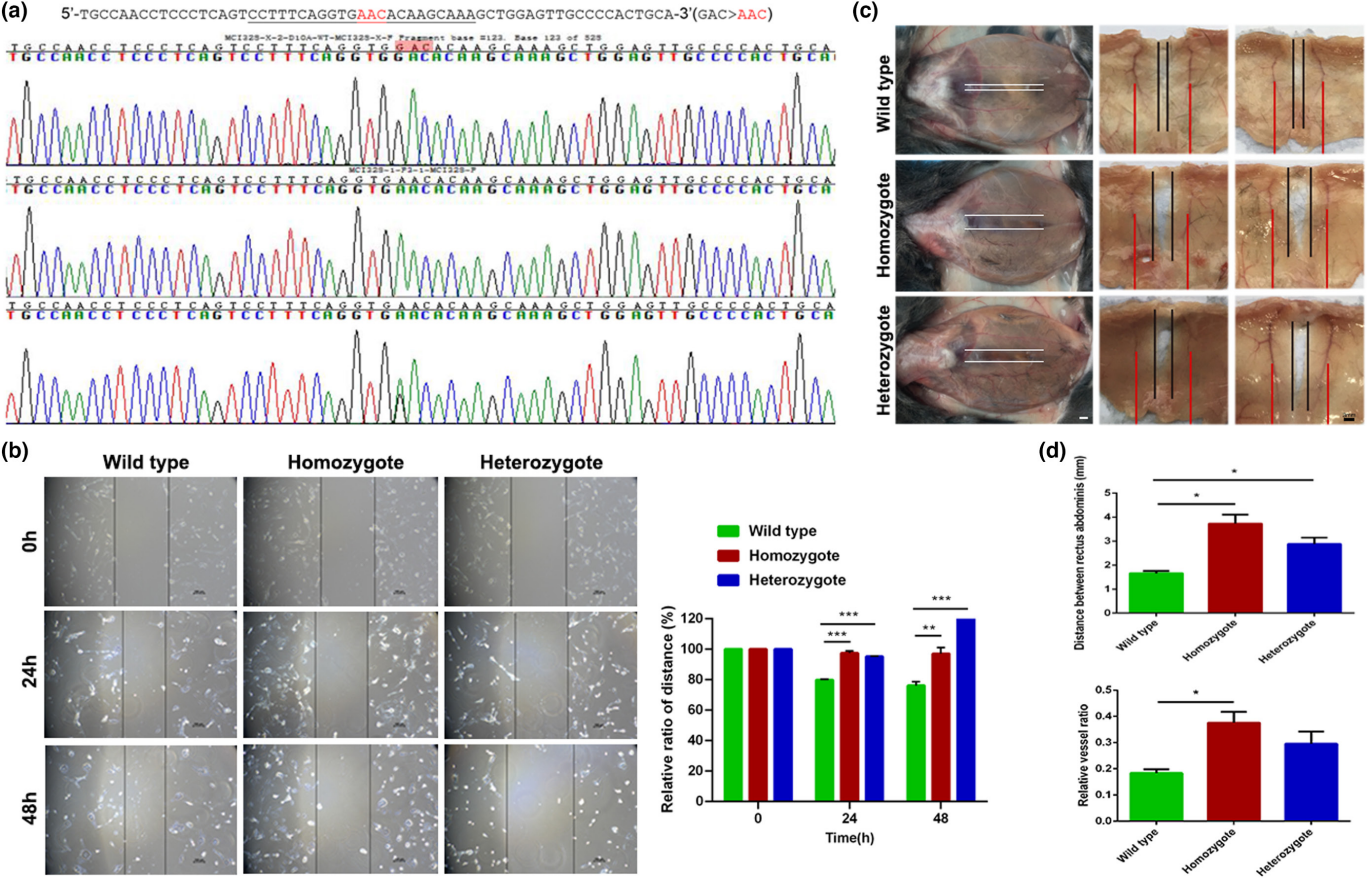
Construction and phenotypic observation of the mouse model of corresponding *FLNA* mutation and the cell migration assay. (a). Construction of the corresponding point mutation of *FLNA* (D1474N, GAC to AAC) by CRISPR/Cas9 genome engineering in mice and division into homozygote, heterozygote and WT groups. (b). *FLNA* mutation suppressed the primary cell migration of abdominal muscle in mice. (c, d). Wider gap of rectus abdominis in homozygote and heterozygote groups compared with WT group, reflected by the absolute distance between rectus abdominis (marked white lines) and the relative ratio of the gap distance between rectus abdominis (marked black lines) to the distance between epigastric arteries (marked red lines) on both sides.

To explore the effect of the *FLNA* mutation (c.4420G>A) on the phenotype, we created a C57BL/6 mouse strain with the corresponding homologous mutation of *FLNA* (D1474N, GAC to AAC) by CRISPR R/Cas9 genome engineering. The pups were genotyped by PCR followed by sequence analysis and no prenatal lethality in hemizygous males was observed. The female mice were divided into homozygote, heterozygote and WT groups (Figure 2a). Mutations in FLNA are primarily associated with skeletal dysplasias but no macroscopic features to suggest skeletal defects with mouse models such as micrognathia and other skeletal malformations. We did not observe an obvious macroscopic phenotype of gastroschisis or omphalocele in our model, but when we separated the skin of the abdomen with fine scissors to expose the abdominal muscles, the central gap between the rectus abdominis in the homozygote and heterozygote groups was found to be wider than that in the WT group (Figure 2c). Therefore, we rapidly separated the abdominal muscle and measured the absolute distance between the rectus abdominis and found a wider gap in the rectus abdominis in the homozygote and heterozygote groups than in the WT group (Figure 2c,d, *p* < 0.05). Furthermore, we evaluated the relative ratio of the central gap to bilateral epigastric artery spacing, and the ratio in the homozygous group was higher than that in the WT group (Figure 2c,d, *p* < 0.05). To gain insight into the generated phenotype and mechanism regulated by *FLNA* mutation, primary cells of abdominal muscle were separated and cultured in each group. A wound healing assay was performed to examine the migratory ability of abdominal muscle cells. The results showed that homozygous and heterozygous mutations suppressed primary cell migration compared with the WT group (Figure 2b, *p* < 0.05).

Therefore, combining the clinical symptoms of fetuses, the results of WES in the family members, the wider central gap between rectus abdominis and the slower muscle cell migration in *FLNA* mutation mice, we considered the diagnosis of MNS induced by the mutation of *FLNA* (c). To our knowledge, MNS disease is extremely rare and has been reported in limited literature. Mutation of *FLNA* was the major cause of MNS. We summarized all the accessible literature about mutation of *FLNA* in MNS, and the details are listed in Table 1. There were 32 cases of MNS with *FLNA* mutations, including 22 cases on exon 22, 7 cases on exon 3 and 1 case on exon 7, and exon 28 with not applicable, respectively. The most common substitutions of amino acids are p. Ala1188Thr and p. Ser1199Leu caused by missense variants c.3562G>A and c.3596C>T on exon 22. Moreover, most patients have facial, skeletal and other organ malformations, especially the male fetus who died in the embryonic or perinatal period. All the male fetuses showed the most obvious malformations, such as omphalocele and renal dysplasia. Here, our proband (III.2, 12 wks) was discerned of a mutation c.4420G>A on exon 26 of the *FLNA* and exhibited the typical feature of midline structure dysplasia of the abdominal wall for MNS.

**TABLE 1:** Cases of MNS with the *FLNA* mutation from various origins in the published work.

DNA nucleotide change	Location	Protein change	Sex	Age at diagnose	Facial features	Skeletal abnormalities	Other malformations	Year	Reference
c.3562G>A	Exon 22	p.Ala1188Thr	Female	5 unaffected individuals	NA	NA	NA	2003	Robertson et al. (2003)
c.3596C>T	Exon 22	p.Ser1199Leu	Female	6 unaffected individuals	NA	NA	NA	2003	Robertson et al. (2003)
c.3552C>A	Exon 22	p.Asp1184Glu	Female	NA	NA	NA	NA	2003	Robertson et al. (2003)
c.3596C>T	Exon 22	p.Ser1199Leu	Female	17 months old	NA	Yes	Yes	2006	Robertson et al. (2006)
3562G>A	Exon 22	p.Ala1188Thr	Female	14 years old	Yes	Yes	NA	2007	Albano et al. (2007)
c.3776G>A+3777G>T	Exon 22	p.Gly1176Asp	Female	40 years old	Yes	Yes	NA	2010	Santos et al. (2010)
c.3776G>A+3777G>T	Exon 22	p.Gly1176Asp	Male	34 weeks old	Yes	Yes	Yes	2010	Santos et al. (2010)
c.3596C>T	Exon 22	p.Ser1199Leu	Female	26‐years old	Yes	Yes	NA	2010	Santos et al. (2010)
c.3596C>T	Exon 22	p.Ser1199Leu	Male	34 weeks old	Yes	Yes	Yes	2010	Santos et al. (2010)
c.3686A>C	Exon 22	p.Tyr1229Ser	Female	23 years old	Yes	NA	Yes	2010	Foley et al. (2010)
c.1054G>T	Exon 7	p.Gly352Trp	Female	32 years old	Yes	Yes	NA	2010	Foley et al. (2010)
Deletion c.4738_4755+10del28 spanning	Exon 28‐intron 28 junction	NA	Female	4 months old	Yes	Yes	Yes	2010	Foley et al. (2010)
c.622G>C	Exon 3	p.Gly208Arg	Female	4 affected individuals of a family	Yes	Yes	Yes	2015	Parrini et al. (2015)
c.3596C>T	Exon 22	p.Ser1199Leu	Male	34 weeks old	Yes	Yes	Yes	2016	Naudion et al. (2016)
c.3562G>A	Exon 22	p.Ala1188Thr	Female	23‐years old	Yes	Yes	NA	2018	Spencer et al. (2018)
c.3562G>A	Exon 22	p.Ala1188Thr	Male	38 weeks old	Yes	Yes	Yes	2018	Spencer et al. (2018)
c.3578T>C	NA	p.Lys1193Pro	Female	3 affected individuals of a family	Yes	Yes	NA	2020	Oh et al. (2020)
c.622G>C	Exon 3	p.Gly208Arg	Female	3 affected individuals of a family	Yes	Yes	Yes	2021	Riccio et al. (2021)
c.4420G>A	Exon 26	p.Asp1474Asn	Male	12 weeks old	Yes	Yes	Yes	Present	

*Note*: There are 22 cases on exon 22, 7 cases on exon 3 and 1 case on exon 7, and exon 28 with NA, respectively. NA means not applicable.

## 4 DISCUSSION

Filamins (FLNs) are essential actin‐binding proteins that regulate intracellular signaling networks and cytoskeletal changes by linking with actin filaments and transmembrane receptors (Chen et al., 2021). The FLN family in humans is composed of three homologous proteins, *FLNA*, *FLNB*, and *FLNC* (Rosa et al., 2019). *FLNA* and *FLNB* are both broadly expressed, whereas the expression of *FLNC* is largely restricted to skeletal and cardiac muscle (Collier et al., 2019; Gardel et al., 2006). These proteins are crucial to mammalian development, and mutations in FLNs result in a wide variety of congenital anomalies including disorders in the brain, bone, muscle, cardiovascular system and many other systems (Kuo et al., 2011; Licht et al., 2019). *FLNA* (gene located on Xq28), which is the most abundant and intensively studied gene, plays a key regulatory role in the remodeling of actin networks, and *FLNA* mutations lead to mitral valve dystrophy (MVD) by impairing cellular signaling and interactions with small GTPases (Le Tourneau et al., 2018). Recently, several studies demonstrated that *FLNA* regulated cell migration and adhesion, and deficiency of *FLNA* resulted in a loss of cell extension and migration (Gad et al., 2012). These findings are similar to our experimental results that showed that the mutation of *FLNA* (D1474N, GAC to AAC) in mice could inhibit the cell migration of abdominal muscle. In addition, *FLNA* is a causal gene of OPDSD, and missense point mutations cause a large range of clinical symptoms in females, such as palatoschisis, palpebral fissures, micrognathia, cardiovascular abnormity, skeletal dysplasia and other organ deformities, through gain‐of‐function effects (Iwamoto et al., 2018).

MNS, a rare disease, is a severe OPDSD that is embryonically lethal in males (S. Robertson, 1993). Our research identified a point mutation of *FLNA* (c.4420G>A) on exon 26 in a family with X‐linked genetic characteristics. We described the family with evidence for two malformed male fetuses, two heterozygous female carriers, and one wild‐type male healthy survivor. Based on the clinical features of fetuses, including hygroma colli, midline structure dysplasia of omphalocele or thoracoceloschisis, abnormal lower limbs and partial facial features, and genetic information with *FLNA* mutation, we considered the diagnosis of the proband as MNS. Here, numerous mutations have been confirmed and involved exons 3, 7, 22 and 26 in Table 1. The vast majority (70%) of MNS patients have pathogenic variants in *FLNA* exon 22, of which two dominant variants are p. Ala1188Thr and p.Ser1199Leu. Rare individuals are identified as having pathogenic variants in exons 3 and 7. In the present research, we found a mutation in exon 26 of *FLNA* for MNS. To date, its genetic pathogenesis remains elusive.

Therefore, we further explored the possible pathogenesis with a mouse strain with the *FLNA* mutation (c.4420G > A). The anatomical results observed in female mice with homologous mutation of *FLNA* (D1474N, GAC to AAC) showed that the gap distances of rectus abdominis in the homozygote and heterozygote groups were wider than those in the WT group. This phenotype might be a precursory feature leading to midline structure dysplasia of the abdominal wall in MNS due to a lack of cell motility and membrane stability through *FLNA* mutation. However, the mouse model did not show the typical phenotype of omphalocele or thoracoceloschisis. The reason may be the differences in abdominal wall thickness and tension among different species. The weakening of muscle cell migration and dysplasia of the abdominal midline structure have not developed to the degree of omphalocele or thoracoceloschisis. On the other hand, there may also be other genes involved that together induced the more severe omphalocele or thoracoceloschisis. Lian et al. observed defects in rib and sternum midline closure leading to thoracoabdominal schisis in *FLNA* + *Fmn2* knockout mice. The results showed that null *FLNA* mice had a thinner ventral body wall than WT mice and that thoracic muscle formation defects, as well as the ventral body wall, were even thinner and defects were more severe in null *FLNA* + *Fmn2* mice (Lian et al., 2017). Therefore, the midline defect may be collectively caused by the combined deletion of *FLNA* and other genes. The specific mechanism in our model still needs to be further explored and studied.

Primary studies considered midline structure dysplasia of the abdominal wall, such as omphalocele or thoracoceloschisis, to be a congenital abdominal wall defect during embryonic development, which is usually not associated with chromosomal abnormalities or genetic mutations (Chircor et al., 2009; Christison‐Lagay et al., 2011). However, recent studies have gradually reported variants of unknown significance (VOUS) and CNVs of pathogenic genes in patients with omphalocele (Kruszka et al., 2015; Shi et al., 2021). The majority of omphalocele patients have other congenital malformations involving the heart, kidney, bone, and nerve (Lupo et al., 2019). Katz et al. screened coding and conserved noncoding regions of *PITX2* for mutations in 209 patients with omphalocele, and a three‐nucleotide deletion was found in the 3′UTR in one case of omphalocele with additional VATER‐like anomalies (Katz et al., 2004). *PITX2* knockout mice have been shown to fail to close the ventral wall, and heterozygotes display variable findings of patent umbilical rings or failure of ventral body wall closure (Gage et al., 1999; Kitamura et al., 1999). Hauptman et al. identified several candidate gene mutations (homozygous mutations in *FAM171B* and *ABCA1*) for gastroschisis by whole‐exome analysis in two patients who presented with myelomeningocele and gastroschisis at birth (Hauptman et al., 2018). In addition, a study demonstrated that a missense mutation occurring in exon 5 of *FLNA* resulted in omphalocele with other congenital malformations (S. P. Robertson et al., 2003). In our case, two fetuses with midline structure dysplasia in this family were highly suspected to be caused by genetic factors, and WES identified a mutation in exon 26 of *FLNA*, which might provide new evidence for the genetic etiology of this dysplasia phenotype.

In conclusion, our study identifies the mutation of *FLNA* (c.4420G>A) on exon 26 as a new pathogenic locus for MNS. Furthermore, we created a mouse model with a point mutation of *FLNA* (GAC to AAC) to further explore the potential mechanisms. Our experimental results indicate that the *FLNA* mutation in mice is related to the abnormal development of a wider central gap of the abdominal wall since the suppressed migration of abdominal muscle cells, as we observed midline structure dysplasia in the two fetuses in the family. Therefore, the mutation of *FLNA* (c.4420G>A) plays an important role in cell motility for the abnormal abdominal wall phenotype of MNS. This description of our finding for the mutation c.4420G > A provides more evidence for clinical diagnosis and genetic counseling of families with these disorders.

## AUTHOR CONTRIBUTIONS

JX and PY designed and supervised the experiments. XL, ZY, JZ, JC, NC and XJ performed the experiments and analyzed the data, and XL wrote the manuscript. JX and QW provided some theoretical and experimental guidance.

## CONFLICT OF INTEREST STATEMENT

The authors have no conflict of interest.

## ETHICS STATEMENT

The study was in accordance with the Declaration of Helsinki and national guidelines. Informed consent was obtained from the participants or their legal guardians.

## Data Availability

The data that support the findings of this study are available on request from the corresponding author. The data are not publicly available due to privacy or ethical restrictions.
